# Prophylactic tocilizumab to prevent cytokine release syndrome (CRS) with teclistamab: A single-center experience

**DOI:** 10.1038/s41408-023-00963-y

**Published:** 2023-12-20

**Authors:** Sara A. Scott, Ellen M. Marin, Kathryn T. Maples, Nisha S. Joseph, Craig C. Hofmeister, Vikas A. Gupta, Madhav V. Dhodapkar, Jonathan L. Kaufman, Sagar Lonial, Ajay K. Nooka

**Affiliations:** Department of Hematology and Medical Oncology, Winship Cancer Institute, Emory University School of Medicine, Atlanta, GA USA

**Keywords:** Myeloma, Myeloma

**Dear Editor**,

Teclistamab (Tecvayli®) is a first-in-class T-cell redirecting bispecific antibody that targets CD3 on the surface of T-cells and B-cell maturation antigen (BCMA) on plasma cells [1]. Teclistamab was approved by the FDA for patients with relapsed/refractory multiple myeloma (RRMM) after 4 lines of therapy including an immunomodulatory drug (IMID), proteasome inhibitor (PI), and anti-CD38 monoclonal antibody (mAb) based on the results of the phase 1/2 MajesTEC-1 study. Among the 165 patients treated with teclistamab, the overall response rate (ORR) was 63% with 58.8% of patients achieving a very good partial response (VGPR) or better. The median duration of response (DOR) was 18.4 months, median progression free survival (PFS) was 11.3 months, and median overall survival (OS) was 18.3 months [2].

The approved schedule per the package insert for teclistamab is 2 step-up doses at least 48 hours apart followed by the first full dose administration (0.06, 0.3, and 1.5 mg/kg). The most common adverse events were neutropenia (70.9%; grade 3–4: 64.2%) and cytokine release syndrome (CRS; 72.1%; grade 3–4: 0.6%). The median time to onset of CRS was two days (range 1–6) and median duration was 2 days (range, 1–9) [2]. CRS and immune effector cell neurotoxicity syndrome (ICANS) are adverse events of special interest among patients receiving T-cell redirecting therapies. A risk evaluation and mitigation strategy (REMS) program was implemented to effectively deliver teclistamab without compromising on safety. The FDA approval label recommends hospitalization for 48 h after each step-up dose and the first full dose [1]. Our objective was to reduce the incidence of higher-grade CRS with prophylactic administration of tocilizumab to mitigate the risk of CRS and facilitate outpatient administration of teclistamab.

A total of 53 patients were admitted to the Emory University Hospital from December 2022 until August 2023 as suggested by the package insert for teclistamab step-up dosing followed by the first full dose administered at least 48 h apart (0.06, 0.3, and 1.5 mg/kg). Premedications including dexamethasone 16 mg, diphenhydramine 25–50 mg, and acetaminophen 650 mg were administered 30 min prior to each dose. Upon evaluation of our first 15 patients, the median time to CRS from the administration of the first-priming dose was 48 h. We subsequently administered tocilizumab 8 mg/kg IV over an hour (max dose of 800 mg) prophylactically at 44 h (4 h prior to the second step-up dose level) for the next 38 patients. CRS and ICANS were graded per the American Society for Transplantation and Cellular Therapy (ASTCT) criteria and managed according to institutional guidelines [3].

The median age of the patients was 69 years (range, 43–83), with additional baseline characteristics in the prophylactic cohort shown in Table 1. All patients were IMID, PI and anti-CD38 mAb refractory. At a median follow-up of 113 days (range, 3–254), the rate of all grade CRS amongst the entire cohort of 53 patients was 39.6% (21). CRS occurred in 26.3% (10) of patients in the prophylactic tocilizumab cohort, compared to 73.3% (11) of patients who did not. The majority of these events were grade 1 [21.1% (8) vs 66.7% (10)]. Of note, 5 of the 10 patients in the prophylaxis cohort experienced CRS after step-up dose 1 and received tocilizumab treatment rather than prophylaxis. In the prophylactic cohort, 1 patient had grade 2 CRS and another had grade 3 CRS. The patient with grade 3 CRS had 41% circulating plasma cells at the time of teclistamab administration. The median number of tocilizumab doses was 1 (range, 1–3), which includes the prophylactic dose. The median duration of CRS was 1 day (range, 1–3). One dose of steroids was administered for treatment of CRS (in addition to the steroids given with premeds) in the 1 patient who experienced grade 3 CRS but in no additional patients treated with prophylactic tocilizumab. Concurrent ICANS was also decreased with the incorporation of prophylactic tocilizumab (20% vs 5.3%, respectively). All ICANS in the prophylactic cohort were grade 1, occurred with concurrent CRS, and managed symptomatically. The median duration of ICANS was 1 day and resolved with CRS resolution. Additionally, none of the patients in the prophylactic cohort were re-admitted to the hospital within 14 days of discharge.

**Table 1:** Baseline Characteristics.

	No Prophylactic Tocilizumab (*n* = 15)	Prophylactic Tocilizumab (*n* = 38)	MajesTEC-1 (*N* = 165) [2]
Median Age, years, (range)	58 (47–73)	69 (43–83)	64 (33–84)
Male, *n* (%)	13 (86.7)	25 (65.8)	96 (58.2)
Race, *n* (%)			
White	7 (46.7)	18 (47.4)	134 (81.2)
Black/African American	8 (53.3)	16 (42.1)	21 (12.7)
Hispanic	—	3 (7.8)	
Asian	—	1 (2.6)	3 (1.8)
Other	—	3 (7.9)	7 (4.2)
ISS Stage at Diagnosis, *n* (%)^a^			
I	2 (22.2)	14 (36.8)	85 (52.5)
II	2 (22.2)	8 (21.1)	57 (35.2)
III	5 (55.6)	15 (39.5)	20 (12.3)
High Risk Cytogenetics	4 (26.7)	17 (44.7)	38/148 (25.7)
BMPCs^b^			
≤5%		13 (34.2)	
≤30%			111 (67.3)
6–60%		5 (13.2)	
30–60%			31 (18.8)
≥60%			8 (4.8)
61–90%		7 (18.4)	
> 90%		2 (5.3)	
Not Assessable		11 (28.9)	
> 1 Extramedullary Plasmacytoma^b^		14 (37)	28 (17)
Median Prior Lines of Therapy, (range)	6 (4–14)	5 (2–13)	5 (2–14)

^a^Six patients in no prophylaxis cohort and one patient in prophylaxis cohort with whose ISS stage could not be assessed; In MajesTEC-1, three patients with whose ISS not assessed.

^b^Bone marrow plasma cells (BMPCs) and extramedullary disease were not assessed in no prophylactic tocilizumab cohort due to change in practice around the time of prophylactic tocilizumab initiation.

**Fig. 1:**
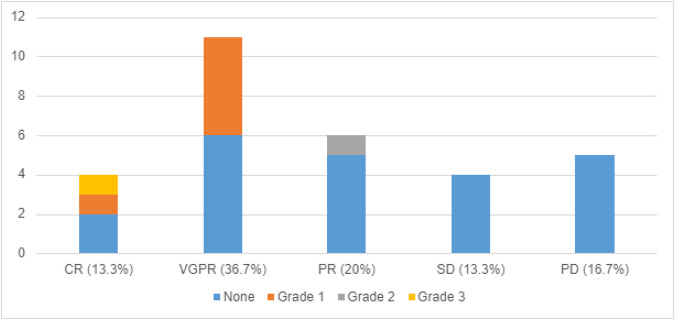
CRS Grade and Disease Response in Patients with Prophylactic Tocilizumab. Myeloma response rate and CRS grade in 30 patients who received prophylactic tocilizumab. Of note, 8 Patients did not have an assessable response due to local follow-up or lack of time between data analysis and the last dose of teclistamab. These patients were not included in the total when calculating the response percentage.

The use of prophylactic tocilizumab resulted in responses comparable to that of the MajesTEC-1 study, with a 70% overall response rate (in patients with assessable responses) and 15 of the 30 assessable patients (50%) achieving a VGPR or better (Fig. 1). Additionally, in this cohort, tocilizumab did not increase the incidence of grade 3 or 4 neutropenia when compared to the MasjesTEC-1 trial, 42.1% vs 64.2% respectively.

Prophylactic tocilizumab prior to the second step-up dose has decreased the incidence and severity of CRS in heavily pre-treated RRMM patients receiving teclistamab. These results are lower in absolute incidence and severity when compared to the MajesTEC-1 trial where CRS was seen in 72% of patients [2]. Prophylactic tocilizumab prevented the usage of steroids, prevented dose delays and prevented readmission to the hospital without impacting response to teclistamab. Moreover, it did not increase the incidence of grade 3/4 neutropenia. In our relatively short period of follow-up, the response rates are similar in this heavily pre-treated population compared to those seen in clinical trials. Continued analysis will include the timing of tocilizumab due to the incidence of CRS after the first teclistamab step-up dose and long-term disease response due to reports of a correlation between an early immune response including CRS during step-up dosing and treatment outcomes [4]. In conclusion, these data support the efficacy of early incorporation of prophylactic tocilizumab through prevention of severe CRS as well as a path to safely administer teclistamab in the outpatient setting.
